# Architecture Capabilities to Improve Healthcare Environments

**DOI:** 10.5812/traumamon.9999

**Published:** 2013-05-26

**Authors:** Ali Ebrahimi, Karim Mardomi, Kasra Hassanpour Rahimabad

**Affiliations:** 1 Trauma Research Center and Plastic Surgery Ward, Baqiyatallah University of Medical Sciences, Tehran, IR Iran; 2 Department of Architecture and Urbanism, Iran University of Science and Technology, Tehran, IR Iran

**Keywords:** Health Facility Environment, Facility Design and Construction, Architecture as Topic

## Abstract

**Background:**

The physical environment of healthcare buildings has great importance in issues such as patient safety, functional efficiency, user satisfaction, healthcare outcomes, and energy and resources consumption.

**Objectives:**

The present study assesses physical environments of Iranian healthcare buildings.

**Materials and Methods:**

This study was performed using a descriptive-analytical method. Data collection was carried out via a written questionnaire.

**Results:**

Based on the findings of this study, "functional efficiency", "user satisfaction", "environmental issues", "patient safety”, “accountability in incidents and disasters", and "flexibility" are regarded as the most issues in the country's hospitals. Also, none of the parameters is "without any problem" and has a "desirable status".

**Conclusions:**

According to the responses, all of the healthcare buildings in this research had flaws in their physical environment, which require attention. Thus, it is necessary to review and pay more attention to the architecture of the country's healthcare buildings.

## 1. Background

The present study aimed to investigate the current architecture status of the country's healthcare structures. Various studies have previously been performed to investigate the physical environment of Iran’s healthcare buildings either directly or indirectly. Initially, in order to commence the present study, a framework was formulated for realms related to the architecture of healthcare buildings and environments. In brief, it can be said that the architecture and quality of the physical environment of healthcare buildings are influential in:

• Improvement of patient privacy and safety (including hospital infections)

• Functional efficiency

• Patient and staff satisfaction

• Healthcare results

• Accountability in incidents, disasters and critical conditions

• Consumption of energy and resources

• Adaptability of healthcare buildings to changes and transformations resulting from scientific developments, technology, and regulations

• Reduction of environmental damage (1-12)

Improvement of physical environments in healthcare buildings is a necessity. Evaluations of domestic articles indicate that the effects of physical environments of the healthcare constructions have been hardly noticed. Issues such as patient safety, user satisfaction, accountability in incidents have often been investigated in terms of management strategies, quality of medical and nursing services and medical equipment facilities; however, the role of architecture to improve the above-mentioned factors has gone unnoticed (13-20).

## 2. Objectives

This study intended to investigate the status of physical environments related to healthcare buildings the architectural capabilities that may be used to improve these environments.

## 3. Materials and Methods

The present study was a cross-sectional one carried-out using a descriptive-analytical method. A written questionnaire consisting of two general parts ( the respondent's personal information and assessment of physical environments of hospitals) was used to collect the data. The personal information section included age, educational degree, place of occupation, duration and quality of their profession in healthcare environments. Questions about the physical environment of the hospitals were based on similar studies (1, 3-5, 7, 9-11). This questionnaire consisted of nine general parts: 1) patient safety 2) the process of treatment, recovery, and clinical results 3) user satisfaction: 4) special needs 5) functional efficiency 6) adaptation to developments and changes; flexibility 7) accountability of hospitals in incidents and disasters 8) environmental problems of hospitals; sustainability 9) evaluation of physical spaces. There were 59 questions with five choices (very difficult = 5 points, not difficult at all = 1 point and I have no idea) and nine open-ended questions, to achieve more precise results. To assess the validity and reliability of the questionnaire "experts' judgment" was used where two experts reviewed the questions and content of the questionnaire and after necessary amendments, its reliability was confirmed. To determine the validity and reliability of the questionnaire test-retest method was applied. Such that 8 questionnaires were distributed among the subjects of the study with a time interval of 7 days and the collected data were analyzed using the SPSS software (17^th^ version) and eventually, Coronbach's alpha test was determined to be 86%. 

The research population was determined with respect to the objectives of the study; two main groups were chosen to complete the questionnaire; they included physicians and nurses as the permanent users of healthcare buildings, and architects and designers with at least 10 years of experience in hospital architecture. Faculty members of the country's universities of medical sciences were selected to determine the population of the physicians and nurses that were to complete the questionnaire. This selection was based on the logic that the academic faculty members have been in contact with at least 10 healthcare centers during their education and service; the academic aspect of their profession make the judgment superior. A list of e-mail addresses of faculty members of all the country's medical universities was prepared for sending the questionnaires. From among 1784 collected e-mail addresses 900 e-mails were selected randomly. The web based questionnaire was sent to the 900 candidates. After four months (March 2012 to June 2012), data collection was complete and analysis commenced. Among the 900 sent questionnaires only 97 were filled; thirteen were incomplete and were omitted and 84 questionnaires were used for the final analysis. Network sampling was used to determine the architects' population and among the experts in this field, 10 people filled-out the questionnaire. consequently, more reliable responses and information were collected. In order to perform the statistical data analysis, SPSS software (the 17th version) and descriptive-inferential statistics were applied. Additionally, the open-ended questions were analyzed by the content-analysis method.

## 4. Results 

Participation rate in this research was 10 percent, which was relatively low and this may be because the questioners were sent via internet and there was a relatively high volume of questions. Opinions of 84 participants were analyzed in this study which included 38 females and 46 males with an average age of 44 years old (standard deviation of 8.31) and the average work experience was 18 years (SD 7.78). Moreover, among the respondents 31 participants were nurses, 43 participants were physicians, and 10 were architects or healthcare construction designers; 48 had PhD degrees, 15 were GPs and 21 of them had Master's degrees. Analysis of the responses provided by different groups (analysis and variance test) suggested that there was no significant difference between the insights of various groups (nurses, physicians, and architects) about the status regarding the physical environment of the country's hospitals. In order to be more organized in presenting the results, the findings are represented in seven categories.

### 4.1. Patient Safety 

According to the participants' opinions, "patient comfort" and "hospital infections", were considered as two important issues of the physical environment (Table 1). In the open-ended question, which was provided in order to report other issues of the physical environment affecting patient safety, many issues were mentioned that can be generally classified into the above-mentioned groups. Content analysis of the answers showed more emphasis on the following factors:

• Inappropriate physical structure of the wards regarding location and circulation control (non-segregated paths for patients, families and staffs) which results in the outbreak of hospital infections

• Shortage of in-patient rooms and poor condition of the patient's physical comfort, crowd and noise pollution

• Inappropriate placement of nurse stations, which functions inefficiently (lack of effective observation of patients)

• Inappropriate lighting of internal environment spaces

**Table 1.** Assessment of Physical Environments from Various Aspects

Aspects of the Physical Environment	Weighted Score ^a^	SD	Answers, No.
**Aspects of Patient Safety**			
Hospital Infections	4.00	0.994	82
Medical Errors	3.52	1.038	81
Physical Injuries (Falls etc.)	3.12	1.023	82
Duration of Admission at the Hospital	3.43	1.079	84
Patient's Physical Comfort	4.02	0.905	84
Patient's Sleeping Pattern	3.78	0.884	83
AverageSafety Status	3.64		
**Aspects of User Satisfaction (Individual, Emotional, and Social needs)**			
Path Finding in Hospital	3.68	1.023	81
Appropriate Relationship WithHealthcare Personnel	3.73	1.031	82
Privacy	3.85	0.904	82
Confidentiality of PatientInformation	3.46	1.147	80
Social Contact	3.53	1.062	81
Emotional Contact	3.77	1.010	82
Conforming to Cultural Needs	4.04	0.928	81
**Aspects of User Satisfaction (Special Needs)**			
Special Needs of Children and Infants	3.88	1.044	78
Special Needs of Overweight Patients	3.88	0.939	78
Special Needs of Elderly Patients	4.13	0.933	80
Needs of Patient's Family and Entourages	4.42	0.744	79
Needs of the Hospital Staff	3.95	0.924	78
Average User Satisfaction Status	3.86		
**Aspects of Functional Efficiency**			
Use of Energy	4.06	1.005	82
Use of Resources and Equipment	3.74	1.040	84
Use of Staff and Human Resources	4.05	0.960	80
Use of Space and Building	4.16	0.916	81
Use of Communications, Information and Media (Information Technology)	4.01	0.843	79
Average of Functional Efficiency Status	4.02		
**Aspects of Flexibility**			
Adaption of Physical Environment to Rapid Changes Related to Medical Technology	3.64	1.100	83
Adaption of Physical Environment to Rapid Changes Related to Information Technology	3.70	1.095	84
Adaption of Physical Environment to Rapid Changes Related to Healthcare Methods and Processes	3.64	0.990	84
Adaption of Physical Environment to Rapid Changes Resulted from Regulations and By-Laws	3.46	0.941	83
Average of the Flexibility Situation	3.61		
**Aspects of Accountability of Healthcare Buildings in Incidents and Disasters**			
Capability of the Physical Environment for Crisis Management	3.62	1.096	78
Confrontation With Infections	3.65	0.988	79
Accountability of Medical Emergencies in Critical Conditions	3.53	1.067	80
Accountability of Healthcare Sections in Critical Conditions	3.49	0.941	77
Strength of the Hospital Building	3.78	1.084	81
Average of Accountability in Incidents and Disasters	3.614		
**The Most Important Incidents in Healthcare Buildings**			
Industrial and Road Incidents	3.60	1.109	78
Natural Disasters (Flood, Earthquake, …)	4.00	1.064	77
Chemical, Biological, Nuclear Attacks	3.88	1.178	73
Terrorist Attack	3.50	1.256	72
**Aspects of Environmental Issues**			
Energy Consumption and Management	3.91	0.971	82
Management of Water Supplies	3.71	1.013	84
Hospital Wastes	3.84	0.987	80
Poisonous Wastes	3.84	0.968	81
Application of Renewable Resources and Materials	3.80	0.992	79
Quality of the Air Inside the Building (Internal Air)	3.91	0.971	82
Average Situation of the Environmental Issues	3.835		

^a^Score 5 represents a basic issue and very undesirable situation; and score 1 represents a quite desirable situation

### 4.2. User Satisfaction

Three cases including "needs of patients' entourages and families", "special needs of elderly people", and "accountability to users' cultural needs" are among the important issues of the physical environment; 92% of the participants evaluated the requirements of the patients' entourages and families as a very important issue (Table 1).As a result of the content analysis performed for the open-ended question, based on emphasis of the respondents, the following factors were noted:

• Poor respect for patient privacy from different aspects

• Lack of appropriate landscaping and campus to be used by staff, patients and their entourages

• Lack of suitable space for families and entourages to attend

• Lack of appropriate and defined waiting rooms

• Ignorance of various personal needs

### 4.3. Functional Efficacy

According to the views of the participants of this study, among the five subjects included in this section, 4 were regarded as basic issues of physical environment from the aspect of functional efficacy; they included "use of space and building", "use of energy", "use of staff and human resources", "application of communication, information and media (information technology)" (Table 1). As a result of the content analysis performed for the open-ended question of this section, which was highly emphasized by the respondents, the following factors were noted:

• Waste of energy

• Poor use of modern technologies and lack of necessary infrastructures

• Wide gap between spaces having functional capability

### 4.4. Flexibility and Adaptation to Changes 

None of the topics suggested the flexibility of the physical environment as a basic issue. Yet, among these issues, "adaption of the physical environment to rapid changes related to information technology" has more sensitivity compared to other issues (Table 1). As a result of the content-analysis carried out for the open-ended question in this section, no factors outside of the above-mentioned issues were mentioned.

### 4.5. Performance of Hospitals in Tragedy and Disasters

None of the issues brought about, in this section were considered as a fundamental issue. But, among them "strength of building structure" had more sensitivity than others (Table 1).In the second part of this section, the participants were asked about which incidents they thought could endanger healthcare buildings? Based on the analysis of the answers, "natural disasters" (floods, earthquakes etc) were regarded as the most important factors, which threaten healthcare buildings. As a result of the content-analysis related to the open-ended question of this section, no other factor was noted apart from the above-mentioned issues.

### 4.6. Environmental Problems of Hospitals

The "quality of the air inside the building", "management of water supplies", "hospital wastes", "poisonous wastes" and "application of renewable resources and materials" were among important issues regarding the physical environment of healthcare buildings; however, none of them were seen as a basic issue (Table1)As a result of the content-analysis related to the open-ended question of this section, no factor apart from the above-mentioned issues were suggested.

### 4.7. Zoning of Hospitals Spaces

Table 2 presents the data analysis of the status of physical environments relevant to segregated spaces. Based on the opinions of the participants of this study, in-patient rooms were the most problematic in comparison to other spaces. Among the hospital wards the "emergency department" was significantly evaluated as the most undesirable; 94% of the participants assessed the physical environment of the emergency department as a very important issue. Also, among the public spaces, the physical environment of "waiting rooms" and "parking" were undesirable. As a result of the content-analysis related to the open-ended question of this section, with high emphasis of the respondents, the following factors were noted: 

• Lack of enough parking lots

• Lack of attention to emotional and social needs of patients

• The internal spaces of units, not being appealing, clean or tidy

• Lack of attention to ergonomics in the internal spaces of wards

• No-defined entrances for buildings

• Lack of appropriate waiting spaces

**Table 2.** Assessment of Physical Environment and Segregated Spaces

Physical Environment and Segregated Spaces	Weighted Score ^a^	SD	Answers, No.
**Observation/Healthcare Spaces**			
Patients' Room	3.76	0.910	82
Exam and Healthcare Rooms	3.56	0.931	82
Surgery Rooms	3.49	1.159	78
Imaging Rooms	3.14	1.041	78
Average Situation of Observation/Healthcare Spaces	3.48		
**Hospital Departments**			
Surgery Ward	3.56	1.169	78
ICU	3.47	1.130	81
NICU	3.44	1.168	71
Emergency Department	4.31	0.869	83
Imaging	3.03	1.070	76
Rehabilitation Unit (Physiotherapy etc.)	3.11	1.143	64
Delivery Units	3.71	1.038	70
In-Patient Unit	3.55	0.978	83
Average Situation of Hospital Units	3.522		
**Public Spaces **			
Entrance/Lobby	3.27	1.236	84
Waiting Spaces	3.85	1.081	84
Corridors	3.32	1.088	84
Parking	3.99	1.160	82
Campus	3.50	1.227	84
**Average Status of Public Spaces**	3.586		

^a^Score 5 represents a basic issue and very undesirable situation and score 1 represents a quite desirable situation

## 5. Discussion

**Figure 1.**
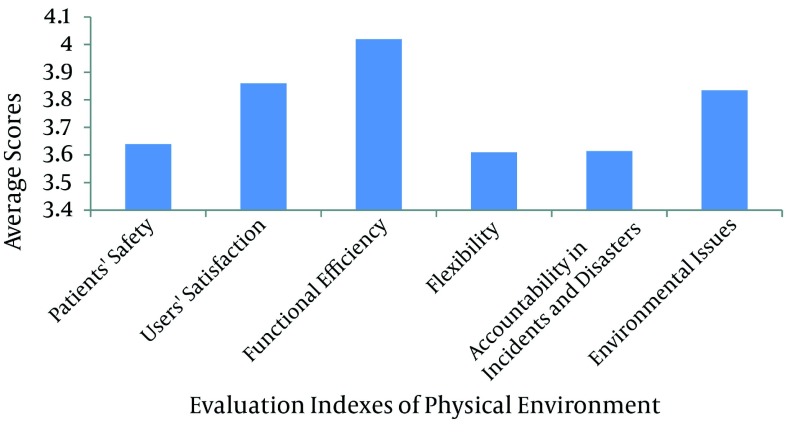
Comparison of Evaluation Indices of Physical Environments. Score 5 represents a very undesirable situation

According to the data analyses, none of the addressed parameters, regarding the physical environment of healthcare buildings was "problem-free" or had a "desirable condition". Based on the respondents' opinions in this research, all the factors mentioned, required more attention and concern. Therefore, it is necessary to review the architecture of the country's healthcare buildings. Figure 1 shows a comparison of various indexes in evaluating the physical environment of healthcare buildings.

**Figure 2.**
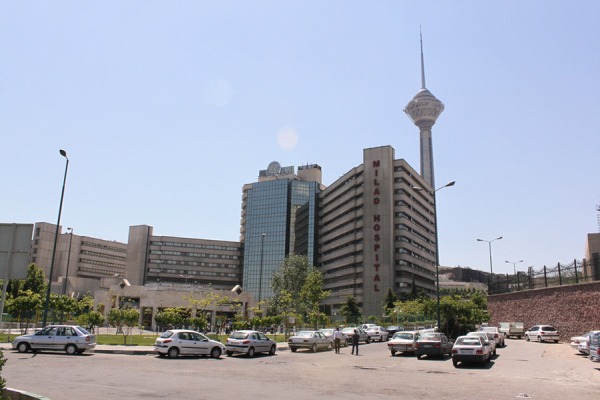
Milad Hospital, Tehran- Note Lack of Landscape

**Figure 3.**
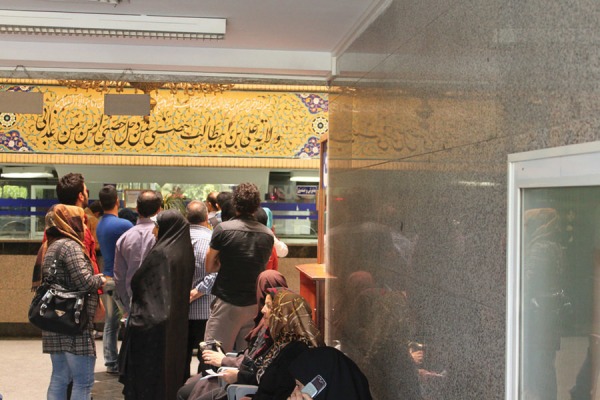
Alghadeer Hospital, Tehran-Emergency Department-Note Disproportion Between the Reception Space and Referrals

According to the findings of this study, "functional efficiency", "user satisfaction", "environmental issues", "patients' safety", "accountability in incidents and disasters", and "flexibility" are considered as the most important issues of the physical environments of the country's hospitals, respectively. As a result of the data analysis of the open-ended questions posed to determine other existing issues (apart from the cases mentioned in this questionnaire), the respondents emphasized addressing the following factors:

• Aging of buildings

• Lack of appropriate relationship between various units (far distances between the related wards)

• Non-standardized space (area and height of ceiling)

• Inefficient workplace for the staff

• Addition of new units (departments) to the old structure which causes disturbance in performance and confusion

• Lack of landscape (Figure 2)

• Disproportion between the spaces and referrals (Figure 3) 

• Disturbing noises and noise pollution resulting from streets and surroundings

In general, comprehensive studies on all the issues of physical environments of the country's healthcare buildings are sparse. Many of the findings in the present study are in accordance with other existing studies. For example, the necessity of improving the safety and security of the physical environment of hospitals for crisis and disasters (16, 17), decrease of noise pollution and promotion of the environmental conditions for patient comfort and sleep (13, 19), improvement of physical environment of hospitals for elderly people's needs (15), patient satisfaction (14), patient's safety and hospital infections (20) and issues of the emergency department physical environment (21) are important. Another considerable point in the present study was the participants' (subjects) attitudes about the emergency room; which had been emphasized as the most challenging unit (ward) in the hospital structure. This issue has been given priority in the twenty-year outlook of the comprehensive scientific healthcare plan of the state's macroeconomic policy. One of the other issues identified in the present study, which is now being pursued at the national level, is "patient safety". Also, proceedings such as strengthening the hospitals for events like earthquakes, forming crisis committees and planning in order to confront incidents, as well as using modern technologies of health information are among the other executive actions in the country that can contribute to address the issues of the physical environment in healthcare buildings. According to the findings of this study, dealing with all the indices addressed here and improving them through architectural capabilities are two necessities. Among these, "functional efficacy", "user satisfaction" and "environmental issues" have higher priorities and significance. It is recommended that the architects and other related specialists address the other issues mentioned herein during the planning and designing stage.

## References

[1] Douglas CH, Douglas MR (2005). Patient-centred improvements in health-care built environments: perspectives and design indicators.. Health Expect..

[2] Guenther R, Vittori G (2008). Sustainable Healthcare Architecture..

[3] Joseph A (2006). The Role Of The Physical And Social Environment In Promoting Health, Safety, And Effectiveness In The Healthcare Workplace..

[4] Joseph A, Rashid M (2007). The architecture of safety: hospital design.. Curr Opin Crit Care..

[5] Lawson B, Phiri M, NHS Estates. (2003). The Architectural Healthcare Environment and Its Effects on Patient Health Outcomes: A Report on an NHS Estates Funded Research Project..

[6] Marberry S (2006). Improving Healthcare With Better Building Design..

[7] McCullough C (2010). Evidence-Based Design For Healthcare Facilities..

[8] Schettler T (2006). Toward An Ecological View Of Health: An Imperative For The Twenty-First Century..

[9] Ulrich R (2000). Proceedings Of The 2nd Annual International Congress On Design And Health. Effects Of Healthcare Environmental Design On Medical Outcomes, In Design and Health-The Therapeutic Benefits Of Design.

[10] Ulrich R, Zimring C, Quan X, Joseph A (2006). The Environment's Impact On Stress..

[11] Ulrich RS, Zimring C, Zhu X, DuBose J, Seo H, Choi Y (2008). A review of the research literature on evidence-based healthcare design.. Health Environ Res Design J..

[12] Ulrich R, Zimring C, Quan X, Joseph A, Choudhary R (2004). The Role Of The Physical Environment In The Hospital Of The 21st Century: A Once-In-A-Lifetime Opportunity..

[13] Afkham E, Ghalehbandi M, Salehi M, Kafiantaghi A, Vakili Y, Akhlaghi F (2008). Investigation of Sleep Parameters and Effective Factors on the Sleep Quality of Out-patients' Referring to the Selected Clinics of Rasoul-e Akram Hospital.. J Iran Univ Med Sci..

[14] Ameriun A, Ebrahiminia M, Azizabadi F, Khodami V, Mehrabi T, Heidari S (2010). The Effect of Demographic Features on the Satisfaction of in-patients from Service Provision in Clinics of Nezami Hospitals.. Discipline Manage Stud..

[15] Arab M, Hosseini M, Ranjbar E, Pourreza A, Varmaghani M, Tajvar M (2010). Satisfaction Rate and Factors Effective on the Elderly People's Satisfaction from Admission Services of Tehran University of Medical Sciences.. Hospital..

[16] Daneshmandi M, Amiri H, Vahedi M, Farshi M, Saghafi A, Zigheimat F (2010). Evaluation of the Preparation Level of Confronting with Crises of Flood, Fire, and Storm in the Iranian Selected Hospitals.. Mil Med..

[17] Maleki M, Shojaei P (2007). Preparation of Educational Hospitals of Iran University of Medical Science against Disasters from the Safety View.. Health Manage..

[18] Nasirpour A, Salmani L (2010). The Role and Capabilities of Tehran Hospitals in Development of Tourism Therapy.. Hospital..

[19] Oreyzi HR, Amiri M, Bahadorian P (2012). Noise Psychological Effect in Isfahan Hospitals Environment.. J Mazandaran Univ Med Sci..

[20] Sabahi Beedgoli M, Shahri S, Kebriaee A, Seyedi HR, Sarafraz Z (2012). Patient safety climate in medical centers of Kashan.. J Health Promot Manage..

[21] Soleimanpour H, Gholipouri C, Salarilak S, Raoufi P, Rajaei Ghafouri R, Pouraghaei M (2012). Assessment of patient satisfaction with emergency department services in Imam Khomeini Hospital, Tabriz, Iran.. J Urmia Univ Med Sci..

